# Reaction mechanisms for electrolytic manganese dioxide in rechargeable aqueous zinc-ion batteries

**DOI:** 10.1038/s41598-021-00148-2

**Published:** 2021-10-21

**Authors:** Thuy Nguyen Thanh Tran, Susi Jin, Marine Cuisinier, Brian D. Adams, Douglas G. Ivey

**Affiliations:** 1 Department of Chemical and Materials Engineering, University of Alberta, Edmonton, AB T6G 1H9 Canada; 2 Salient Energy Inc., Dartmouth, NS B3B 1C4 Canada

**Keywords:** Batteries, Batteries, Electrochemistry, Electrochemistry

## Abstract

This study reports the phase transformation behaviour associated with electrolytic manganese dioxide (EMD) utilized as the positive electrode active material for aqueous zinc-ion batteries. Electrochemical techniques, including galvanostatic charge–discharge and rotating ring-disk electrode measurements, and microstructural techniques, using X-ray powder diffraction, scanning electron microscopy, and transmission/scanning transmission electron microscopy, were utilized to characterize the positive electrode at different stages of discharge and charge of zinc-ion cells. The results indicate that, during discharge, a fraction of EMD undergoes a transformation to ZnMn_2_O_4_ (spinel-type) and Zn^2+^ is intercalated into the tunnels of the γ- and ε-MnO_2_ phases, forming Zn_x_MnO_2_ (tunnel-type). When a critical concentration of Mn^3+^ in the intercalated Zn_x_MnO_2_ species is reached, a disproportionation/dissolution reaction is triggered leading to the formation of soluble Mn^2+^ and hydroxide (OH^–^) ions; the latter precipitates as zinc hydroxide sulfate (ZHS, Zn_4_(OH)_6_(SO_4_)·5H_2_O) by combination with the ZnSO_4_/H_2_O electrolyte. During charge, Zn^2+^ is reversibly deintercalated from the intergrown tunneled phases (γ-/ε-Zn_x_MnO_2_), Mn^2+^ is redeposited as layered chalcophanite (ZnMn_3_O_7_·3H_2_O), and ZHS is decomposed by protons (H^+^) formed during the electrochemical deposition of chalcophanite.

## Introduction

Manganese-based oxides, because of their low cost, low toxicity and their relatively high reduction potentials, have received widespread attention since the 1990s in the field of electrochemical energy storage, such as supercapacitors, pseudocapacitors, primary batteries, rechargeable metal-air batteries, and Li-ion batteries (LIBs)^1–4^. The remarkable diversity of atomic architectures has provided manganese (Mn) oxides the ability to accommodate a wide variety of metallic cations. Rechargeable Zn-ion batteries (ZIBs) using a mild aqueous electrolyte offer the potential for a cheaper and safer choice relative to LIBs for stationary energy storage systems. As such, recent efforts have been made to employ Mn oxides as active materials for positive electrodes of ZIBs. Multifarious Mn oxides have been utilized as positive electrodes in ZIBs, including α-MnO_2_ (2 × 2 tunnels)^5–10^, β-MnO_2_ (1 × 1 tunnels)^11–13^, γ- or ε-MnO_2_ (1 × 1 and 1 × 2 tunnels)^14–17^, todorokite (3 × 3 tunnels)^18^, δ-MnO_2_ (layered structure)^19,20^, and other Mn oxides with different oxidation states such as Mn_2_O_3_^21,22^, Mn_3_O_4_^23,24^, and ZnMn_2_O_4_^25^. Manganese dioxides (MnO_2_) used in energy storage devices are generally classified into three categories based on their origin including natural MnO_2_ (NMD), chemical MnO_2_ (CMD), and electrolytic MnO_2_ (EMD)^26^. NMD is the only one obtained from natural ores. It is a mixture of several Mn oxide minerals (up to 20 different types) and has lower and inconsistent performance compared with the other two forms^26^. Between the two synthesis pathways, electrochemical deposition methods are known to be superior to chemical synthesis methods, as the purity and properties of the deposited material are better controlled^27^. Thus, in commercial batteries, EMD is predominantly used and is likely to remain the preferred energy material for the foreseeable future^27^. Given the widespread usage of EMD in existing commercial batteries, along with its low-cost and established supply chains, EMD is worth exploring for less developed energy storage systems such as ZIBs.

Although several studies have been done to investigate Mn oxides, the storage mechanisms with respect to their electrochemical performance in ZIBs remain ambiguous. Generally, most papers report that Zn^2+^ is inserted into both tunneled and layered MnO_2_, but the detailed mechanisms during discharge and the stability of the structures have not been thoroughly examined^28,29^. The literature has also suggested that H^+^ insertion to form MnOOH during discharge may occur solely^9^, simultaneously^28^, or sequentially^29^ with Zn^2+^ insertion. Some studies have suggested that the formation of MnOOH occurs first and is followed by dissolution of highly soluble Mn^2+^ by electrochemical reduction (MnOOH + H_2_O + e^−^  → Mn^2+^  + 3OH^–^)^30^ or chemical disproportionation (2MnOOH → Mn^2+^  + MnO_2_ + 2OH^–^)^9^ and that there is no Zn^2+^ insertion. On the other hand, it has been reported that Mn^4+^ in MnO_2_ is reduced to Mn^3+^ upon electrochemical intercalation of Zn^2+^ and the resulting Mn^3+^ compound is disproportionated into Mn^4+^ and Mn^2+^ (2Zn_0.5_MnO_2_ + 2H_2_O → Zn^2+^  + Mn^2+^  + MnO_2_ + 4OH^–^)^31^.

The purpose of this study is to investigate the mechanisms associated with the use of EMD as the positive electrode in aqueous ZIBs. Cycled batteries are disassembled to characterize the EMD electrodes at different potential stages using X-ray powder diffraction (XRD), scanning electron microscopy (SEM) and transmission/scanning transmission electron microscopy (TEM/STEM). The results are compared with and supported by electrochemical measurements, including galvanostatic charge–discharge and rotating ring-disk electrode tests. The results indicate that the discharge/charge mechanism is quite complex and is likely the reason for such contradictory reports in the past^9,28–31^. Briefly, there is reversible Zn^2+^ intercalation into the tunnels of γ-/ε-MnO_2_ (in EMD) in addition to dissolution/precipitation side reactions.

## Results and discussion

### Electrolytic manganese dioxide

**Figure 1:**
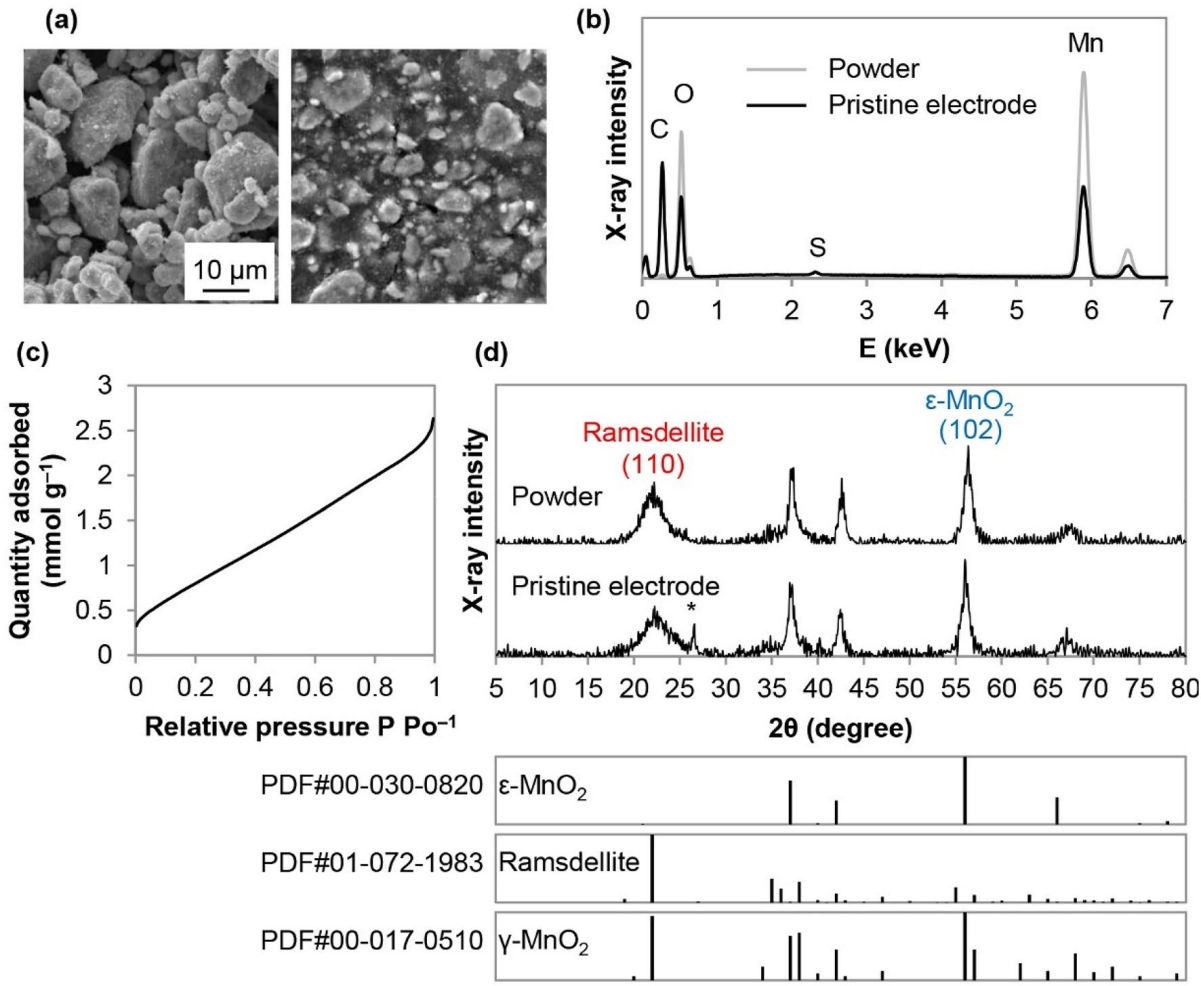
(**a**) SEM secondary electron (SE) images of the EMD powder (LHS) and pristine EMD electrode (RHS). (**b**) EDX spectra from the entire image areas shown in (**a**). (**c**) Nitrogen adsorption isotherm for the EMD powder. (**d**) XRD patterns, along with powder diffraction files (PDFs) for ε-MnO_2_, ramsdellite, and γ-MnO_2_. The asterisk indicates a graphite peak from the current collector.

The morphology and composition of the EMD powder and pristine electrodes are shown in Fig. 1a and b. SEM images show that the EMD particle size, prior to electrode fabrication, is generally ≤ 10 μm. It is clear from the EDX data that the composition of the pristine electrode is similar to that of raw EMD; the carbon peak in the electrode is from carbon black and/or the graphite substrate used as a current collector for these electrodes. The Brunauer–Emmett–Teller (BET) specific surface area of EMD powder, determined from the nitrogen adsorption isotherm (Fig. 1c), is ~ 72 m^2^ g^–1^. As shown in Fig. 1d, the XRD patterns from the EMD powder and the electrode are quite similar. The peaks can be indexed to a mixture of three phases: akhtenskite (ε-MnO_2_), ramsdellite, and nsutite (γ-MnO_2_). These MnO_2_ polymorphs have MnO_6_ octahedra as the basic building block, which are assembled by sharing edges and/or corners to form tunneled structures. Ramsdellite has a (1 × 2) tunnel structure, while both γ-MnO_2_ and ε-MnO_2_ have intergrown (1 × 1) and (1 × 2) tunnels from pyrolusite and ramsdellite, respectively. In general, ε-MnO_2_ has a more disordered structure than γ-MnO_2_^32^. Rietveld refinement, using Jade Pro software, was utilized to determine approximate EMD phase compositions. The EMD powder is composed of about 53% ε-MnO_2_, 34% ramsdellite, and 13% γ-MnO_2_. The result is in agreement with other reports in the literature indicating that EMD usually contains large amounts of ε-MnO_2_ (~ 50%) and ramsdellite (~ 40%), with a smaller amount of γ-MnO_2_ (~ 10%)^33^. Many of the XRD peaks for the three phases overlap, but the peaks at 22.1° and 56.1° are likely primarily from ramsdellite and ε-MnO_2_, respectively.

### Galvanostatic cycling of EMD electrodes

**Figure 2:**
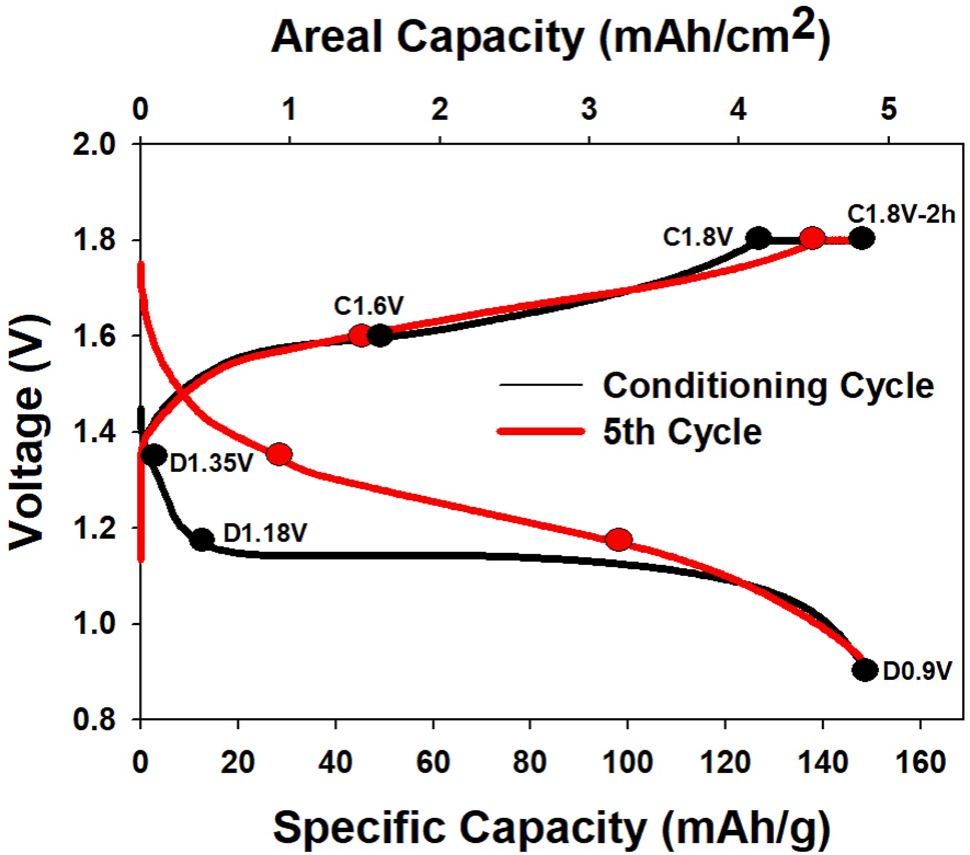
Discharge/charge voltage profiles for the first and fifth cycles of a Zn||EMD cell showing where electrodes were extracted for characterization.

Figure 2 shows voltage profiles with points indicating where EMD electrodes were extracted and characterized. For cycling the zinc-ion Zn||EMD cells, a constant current (CC) was used for discharge and a constant current–constant voltage (CC–CV) protocol was used for charge in the voltage window of 0.9 V to 1.8 V. The typical specific capacity for these cells was roughly half that of the theoretical value of 308 mAh g^–1^ for a one electron reaction (Fig. 2), indicating that there is a significant amount of inaccessible active material and only ~ 0.5 e^−^ is actually transferred. This is likely due to the mixture of phases in EMD (Fig. 1) which lacks long-range order and disrupts diffusion of Zn^2+^ in the tunnels of ramsdellite, ε-MnO_2_, and γ-MnO_2_. The specific capacity is well known to be dependent on the crystallographic phase of MnO_2_ in zinc-ion cells^34^. The specific capacity is also very dependent on other parameters such as particle size and the effective surface area, which is associated with pore size distribution and pore volume^35,36^. Higher surface areas can enhance the battery capacity; however, the dissolution rate is also increased^36,37^. For practical battery systems, areal capacity is a more relevant metric that depends on the thickness and porosity of the electrode coating. Although the specific capacity is relatively low for EMD, the high loading electrodes used in this study result in areal capacities of 4–5 mAh cm^–2^.

For the characterization of electrodes discussed below, cells were stopped at three points during discharge (1.35 V, 1.18 V, and 0.9 V) and three points during charge (1.6 V, 1.8 V, and after the CV step at 1.8 V) for the first conditioning cycle and the fifth cycle. The CV step typically lasted for 2 h which is why it is labelled C1.8 V-2 h.

Several studies have increased the charging potential to 1.9 V^20,38,39^ and even to 2 V^18,40^; however, this will result in the oxygen evolution reaction (OER) at the positive electrode. Therefore, galvanostatic charge–discharge (GCD) tests with the charging potential limited to 1.8 V, followed by a constant voltage period, can alleviate the OER issue.

### Dissolution of Mn^2+^

**Figure 3:**
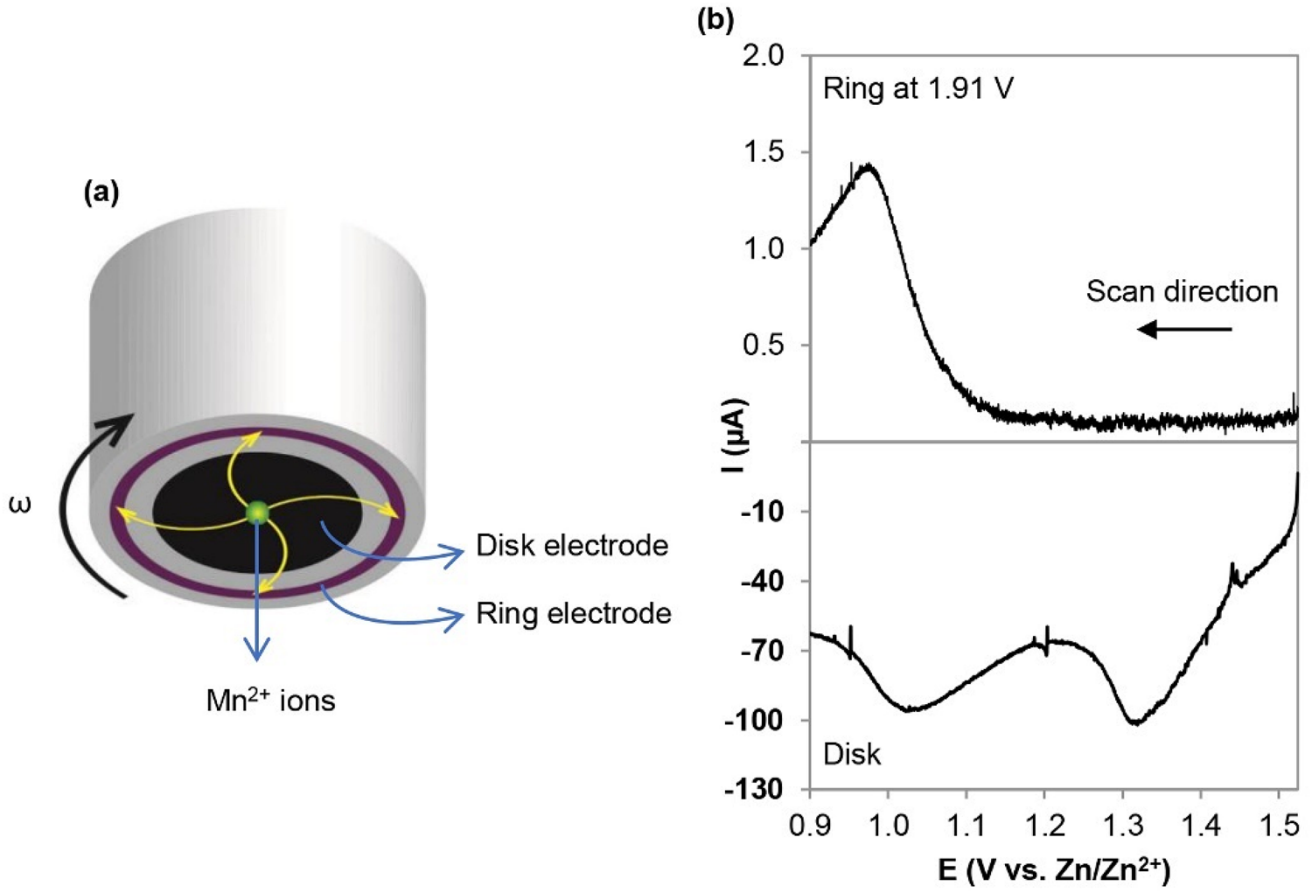
RRDE analysis of EMD. (**a**) Schematic diagram of RRDE tip. (**b**) RRDE profiles of EMD recorded at 1 mV s^–1^ in an Ar-saturated 1 M ZnSO_4_ solution, stirred at 900 rpm with the Pt ring maintained at 1.91 V versus Zn/Zn^2+^.

A rotating ring disk electrode (RRDE) was used to study the electrochemical reactions of the EMD electrodes, specifically to detect soluble Mn^2+^ species formed during discharge. Linear sweep voltammetry (LSV) was performed on the disk where generated products (in the form of soluble Mn^2+^ here) are swept outward by convection caused by rotation and can be detected electrochemically at the ring (Fig. 3a). The ring electrode was held at constant voltage (1.91 V vs. Zn/Zn^2+^) to oxidize the soluble Mn^2+^ to MnO_2_ (Mn^2+^  + 2H_2_O → MnO_2_ + 4H^+^  + 2e^−^). In Fig. 3b, two clear reduction peaks for EMD on the disk electrode are observed, although the current on the ring electrode only increases after scanning below ~ 1.1 V indicating that Mn^2+^ is formed/dissolved at lower voltages during the discharge process. Several Mn^2+^ dissolution pathways have been proposed in the past^9,30,31^, all accompanied with the formation of hydroxide species; however, the disproportionation reaction is observed here for EMD. As the Mn^4+^ in MnO_2_ is reduced to Mn^3+^ upon electrochemical intercalation of Zn^2+^, the resulting Mn^3+^ compound is disproportionated into Mn^4+^ and Mn^2+^ (2Zn_0.5_MnO_2_ + 2H_2_O → Zn^2+^  + Mn^2+^  + MnO_2_ + 4OH^−^)^31^. In the next section, through XRD and selected area electron diffraction (SAED) analysis, both intercalated Zn_x_MnO_2_ and spinel phase hetaerolite (ZnMn_2_O_4_) are found to form during the discharge process. As shown in Figure S1, hetaerolite formation is independent of potential and is stable during discharge, while intercalated Zn_x_MnO_2_ is unstable due to the Jahn–Teller effect and undergoes disproportionation.

### Solid phase identification

**Figure 4:**
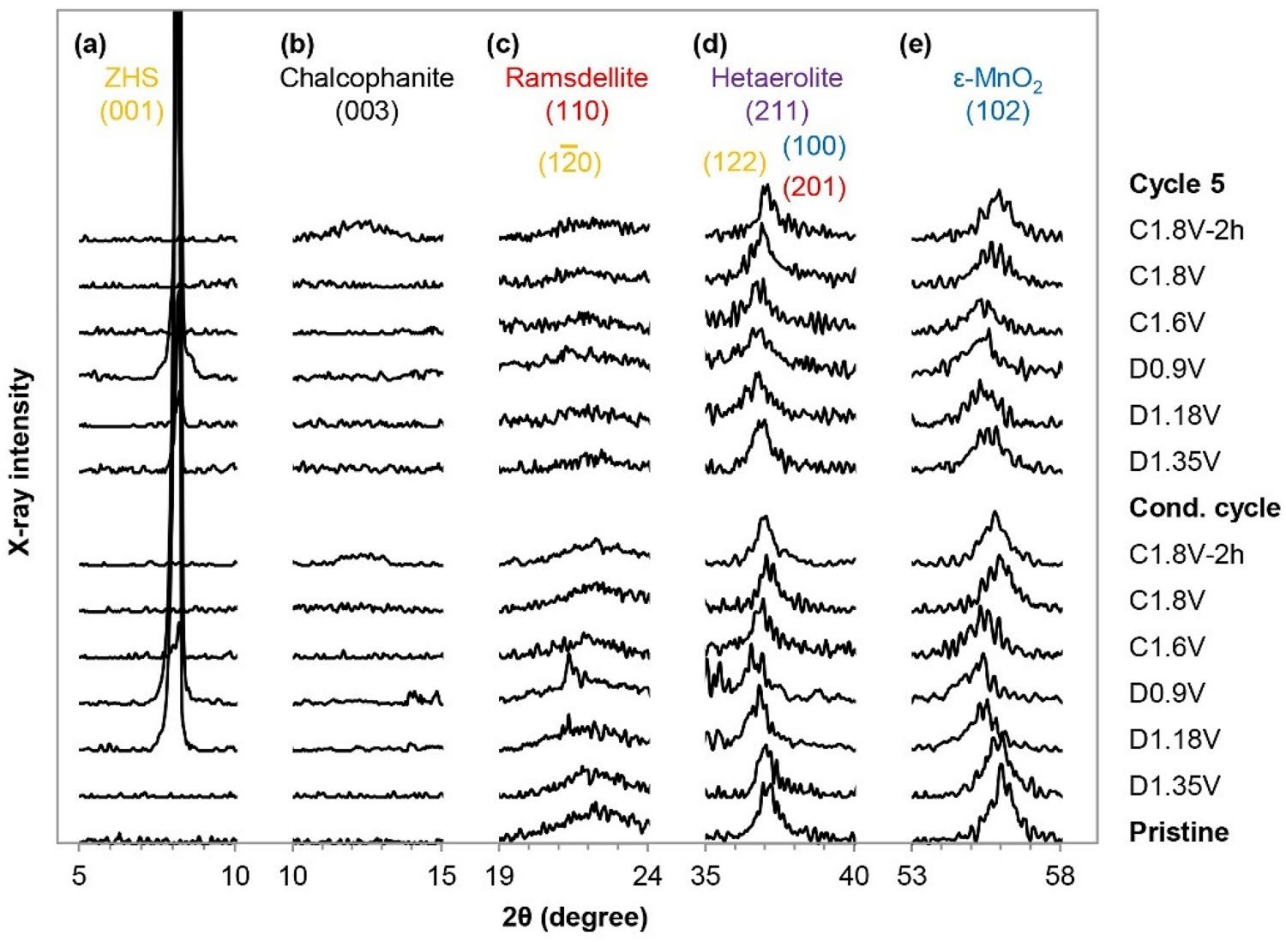
XRD patterns of EMD electrodes at different potentials during GCD tests with the conditioning cycle at 0.5 mA cm^–2^ and the 5th cycle at 1 mA cm^–2^. The chosen peak ranges are representive of (**a**) ZHS, (**b**) chalcophanite, (**c**) ramsdellite, (**d**) hetaerolite, and (**e**) ε-MnO_2_.

To examine the reaction mechanisms further, all EMD electrodes were analyzed by ex-situ XRD (Figure S2). In an effort to make the analysis clearer and to provide evidence for the overall scheme, the patterns are separated into different angular ranges in Fig. 4.

One of the most interesting discoveries of this study lies in the fact that ramsdellite and ε-MnO_2_ in EMD behave differently during cycling. For both the conditioning and the 5th cycles, the major peak ((102) plane) for ε-MnO_2_ at 56.1° shifts to a lower angle of 55.1° at full discharge (0.9 V) (Fig. 4e). In fact, all other major ε-MnO_2_ peaks ((100), (101) and (110), originally at 37.1°, 42.5° and 67.0°, respectively) shift to lower angles during discharge. The peak shifts indicate an increase in both the a and c lattice parameters leading to expansion of the lattice structure. The *a* lattice parameter increases from 0.2797 to 0.2830 nm (1.18% increase), while the *c* lattice parameter increases from 0.4457 to 0.4543 nm (1.93% increase), with an overall cell expansion of 4.36%. The peaks shift back to their original positions after charging, meaning that the structure of ε-MnO_2_ can expand and collapse reversibly to accommodate Zn^2+^ ions. Since larger tunnels are favorable to store and transfer metallic ions without steric hindrance^41^, it is postulated that the (1 × 1) part of the intergrown network is less likely to participate in Zn^2+^ insertion while keeping the structure intact. The XRD results indicate the insertion of Zn^2+^ into the tunnels of ε-MnO_2_ (and possibly for γ-MnO_2_), which seems to occur gradually as the potential varies from open circuit potential to 0.9 V and is reversible during charging. The reaction can be written as follows:

Reaction 1 $$\upgamma ,\varepsilon {\text{ - MnO}}_{2} + {\text{xZn}}^{2 + } + 2{\text{xe}}^{ - } \leftrightarrow \upgamma ,\varepsilon {\text{ - Zn}}_{{\text{x}}} {\text{MnO}}_{2}$$

**Figure 5:**
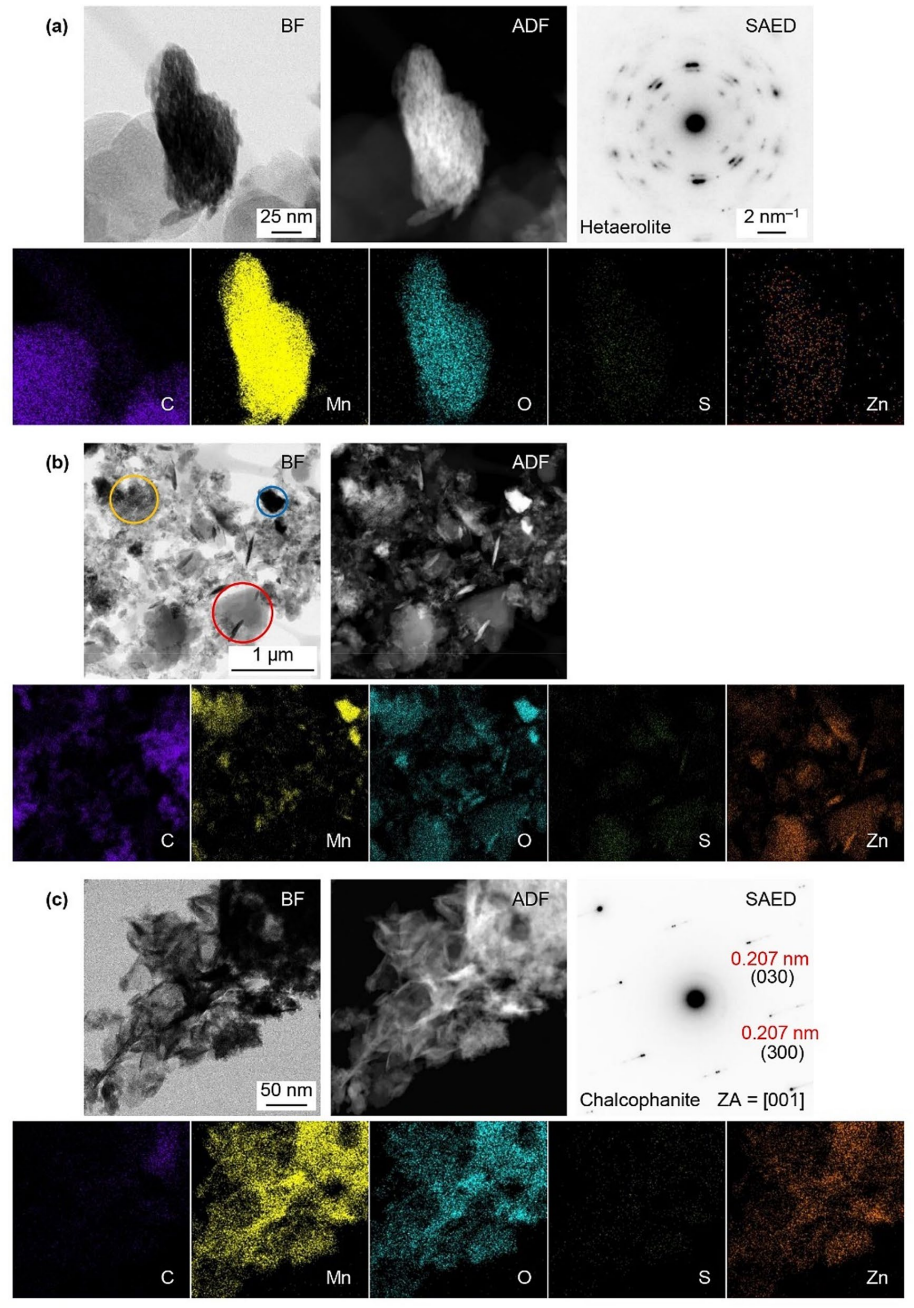
STEM bright field (BF) and annular dark field (ADF) images, EDX maps and SAED patterns from the EMD electrodes at different stages during cycling. (**a**) Electrode discharged at 1.35 V during the conditioning cycle, (**b**) electrode charged at 1.8 V during the 5th cycle, and (**c**) fully charged electrode held for 2 h at 1.8 V during the 50th cycle.

The XRD results were not able to confirm the formation of hetaerolite (with the main peak at ~ 36.5°) during discharge due to interference from both MnO_2_ and ZHS peaks (Fig. 4d). However, the presence of hetaerolite was confirmed through TEM/STEM analysis (see subsequent paragraphs, Fig. 5).

The major peak for ramsdellite at 22.1° (Fig. 4c) continuously decreases in intensity during discharge and does not recover after subsequent cycles. Instead, a new, broad peak emerges at ~ 12° (Fig. 4b) after charging and maintaining the potential at 1.80 V for 2 h. This peak corresponds to a hydrated Mn-Zn oxide phase known as chalcophanite (ZnMn_3_O_7_·3H_2_O). Thin film-XRD (TF-XRD) was used to examine the surface of the fully charged electrode and the new peak is shown with a better signal-to-noise ratio and higher intensity compared with the powder XRD results (Figure S3). Instead of having a tunnel structure like the starting material, chalcophanite is analogous to birnessite with a layered MnO_6_ octahedral structure containing ZnO_6_ interlayers^42,43^. The transformation of the tunneled structure or the electrodeposition of Mn^2+^ to form a layered birnessite-like structure has been reported previously^31,44,45^. In addition, Post et al. showed the structural similarities between the chalcophanite, anhydrous chalcophanite, and hetaerolite^46^. It is postulated that the dissolved Mn^2+^ ions formed during discharge are redeposited during charge, converting hetaerolite to chalcophanite. The reaction can be written as follows:

Reaction 2 $${\text{ZnMn}}_{2} {\text{O}}_{4} + {\text{Mn}}^{2 + } + 6{\text{H}}_{2} {\text{O}} \to {\text{ZnMn}}_{3} {\text{O}}_{7} \cdot 3{\text{H}}_{2} {\text{O}} + 6{\text{H}}^{ + } + 4{\text{e}}^{ - }$$

In Fig. 4a, the peak located at 2θ = 8.1° increases in intensity as the potential decreases from 1.18 to 0.9 V and is assigned to zinc hydroxide sulfate (ZHS). The best fit for the ZHS phase is Zn_4_(OH)_6_(SO_4_)⋅5H_2_O. The proposed pathway of Mn^2+^ dissolution (2Zn_0.5_MnO_2_ + 2H_2_O → Zn^2+^  + Mn^2+^  + MnO_2_ + 4OH^–^) results in the formation of hydroxide ions (OH^–^) which increase the local pH and lead to the undesired formation of layered double salts with a composition that is dependent on the electrolyte salt. In the present work, where aqueous zinc sulfate electrolyte is used, the precipitation product is ZHS (Reaction 3). ZHS has a layered structure consisting of stacked Zn(OH)_2_ sheets with the interlayer spaces filled with ZnSO_4_ as well as water molecules (Zn_4_(OH)_6_(SO_4_)⋅z H_2_O or also known as ZnSO_4_.3Zn(OH)_2_⋅z H_2_O, with z = 1/2, 1, 3, 4, and 5)^47^. The interlayer distance varies significantly depending on the hydration state, but is typically in the range of 7–11 Å^48^.

Reaction 3 $$4{\text{Zn}}^{2 + } + 6{\text{OH}}^{ - } + {\text{SO}}_{4}^{2 - } + {\text{zH}}_{2} {\text{O}} \to {\text{Zn}}_{4} \left( {{\text{OH}}} \right)_{6} \left( {{\text{SO}}_{4} } \right) \cdot {\text{zH}}_{2} {\text{O}}$$

The Pourbaix diagram (Figure S4) clearly indicates that pH changes can lead to the formation of ZHS, which significantly increases the battery internal resistance^49^. However, it is admittedly difficult to detect the pH change in the vicinity of the electrode surface since ZHS acts as a buffer when forming and dissolving in the presence of OH^–^ and H^+^, respectively. The ZHS peak intensity decreases during charging and disappears at 1.8 V and ZHS formation is suppressed during later cycles (Fig. 4a). The disappearance of the ZHS during charge is a direct result of the protons produced from Reaction 2:

Reaction 4 $${\text{Zn}}_{4} \left( {{\text{OH}}} \right)_{6} \left( {{\text{SO}}_{4} } \right) \cdot 5{\text{H}}_{2} {\text{O}} + 6{\text{H}}^{ + } \leftrightarrow 4{\text{Zn}}^{2 + } + {\text{SO}}_{4}^{2 - } + 11{\text{H}}_{2} {\text{O}}$$

Research on the battery reaction mechanisms has been hampered by the fact that the crystal structures for many of the Mn oxide phases are similar and, further increasing the difficulty of analysis, the products forming on the electrodes are often fine-grained, poorly crystalline mixtures. Thus, samples were examined using TEM/STEM to compliment the XRD results. The electrode discharged at 1.35 V during the conditioning cycle is shown in Fig. 5a, including an example of an SAED pattern from the polycrystalline particle shown. The pattern was indexed to hetaerolite (ZnMn_2_O_4_) (Table S1). The EDX maps (Fig. 5a) indicate that the Zn signal overlaps with the Mn and O signals, which confirms that Zn^2+^ is readily inserted into MnO_2_ during the first stage of discharge at 1.35 V. There is also a weak S signal which is residue from electrolyte overlaps with the particle.

STEM images and EDX maps of the EMD electrode after charging to 1.8 V (5th cycle) are shown in Fig. 5b. Three distinct regions are visible: Mn–O regions similar to the pristine EMD (blue circle), Mn-Zn–O regions which are hetaerolite and/or chalcophanite (yellow circle), and Zn–S–O regions which are residual ZHS (red circle). If battery charging is stopped at this stage and the battery is subsequently discharged, ZHS would accumulate and increase the battery internal resistance significantly. These results show that extending the charging time at 1.8 V is necessary to completely remove ZHS.

STEM images and EDX maps for the EMD electrode charged at 1.8 V and held for 2 h are shown in Fig. 5c. The Mn, O, and Zn signals in the EDX maps strongly overlap, while S signal is much weaker. Several SAED patterns were obtained (one example is shown here) and indexed to chalcophanite. According to the EDX analysis of the region in Fig. 5c, the Mn/Zn ratio in chalcophanite is ~ 2.5.

### Morphology changes during discharge/charge

**Figure 6:**
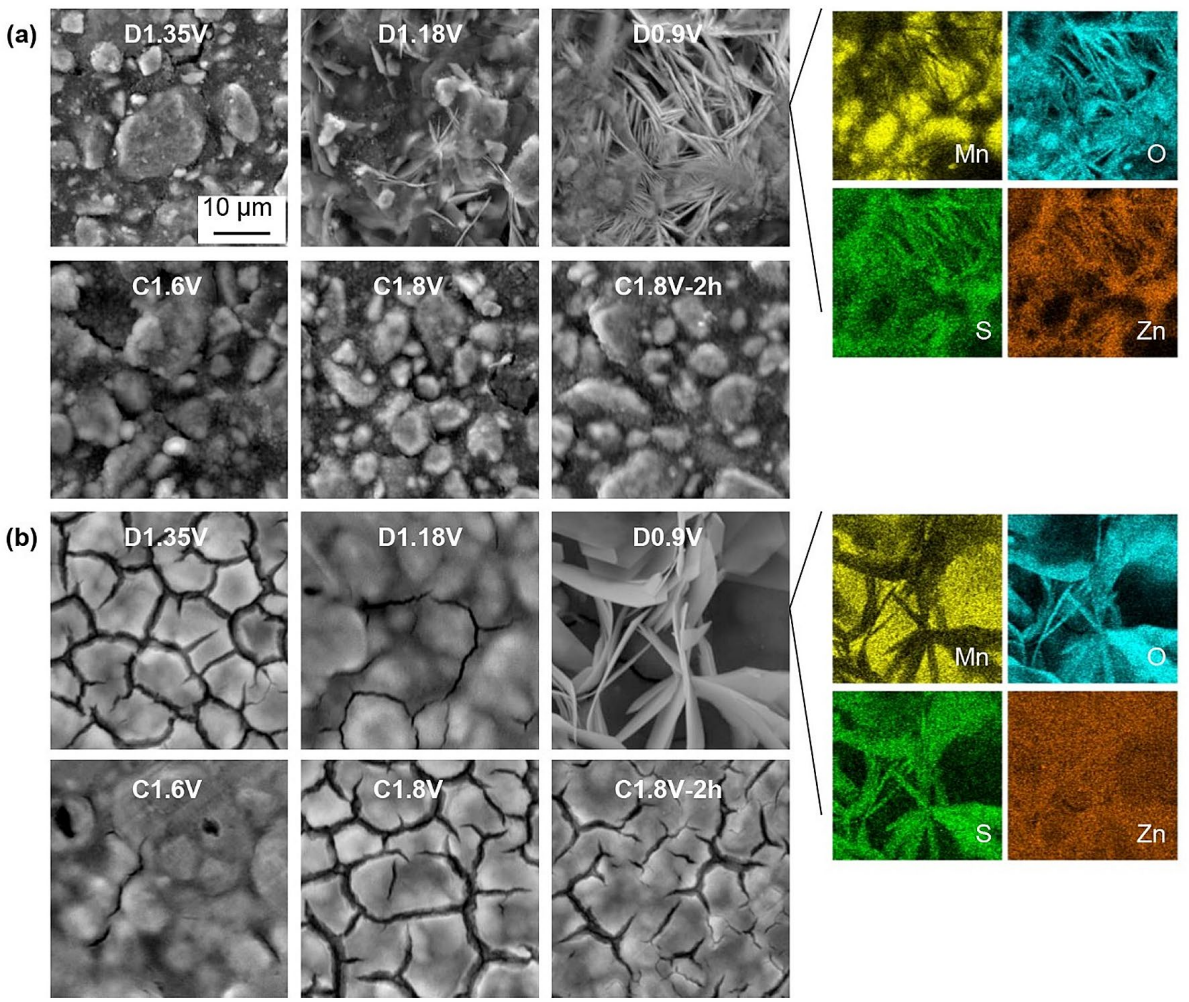
SEM SE images of EMD electrodes at different potentials during cycling tests. (**a**) Conditioning cycle at 0.5 mA cm^–2^ and (**b**) the 5th cycle at 1 mA cm^–2^. EDX maps for the D0.9 V images. Discharge and charge processes are denoted as D and C, respectively.

SEM and EDX analysis, shown in Fig. 6, were used to monitor the morphology evolution of the EMD electrodes during the charge/discharge processes. During the first discharge (Fig. 6a), the electrode surface was gradually covered with ZHS flakes which indicates an increased amount of OH^–^ near the electrode surface at 1.18 V. During charging, the amount of ZHS is reduced and the EMD surface recovers. For the 5th cycle (Fig. 6b), ZHS appears at a potential lower than 1.18 V during discharge. Since a higher Mn^2+^ concentration is already available in the electrolyte, Mn^4+^ dissolution is reduced thereby delaying ZHS formation in subsequent cycles. This is the main reason for adding manganese sulfate (0.1 M MnSO_4_) to the electrolyte^20^. The electrode surface morphology changes drastically compared with the pristine electrode or the initial charge/discharge process. Although ZHS formation is reversible, the EDX maps show that Zn seems to have covered the electrode surface and the Zn signal overlaps with the Mn signal for subsequent cycles rather than just appearing in the ZHS region. The surface of the EMD electrode after 50 cycles is shown in Figure S5, confirming that the electrode surface is covered with a new layered material of Zn–Mn oxide. The XRD results (Fig. 4) showing the formation of chalcophanite after full charge correlate well with the SEM images and EDX mapping (Fig. 6) of the Mn-Zn oxide layer that forms and builds up on the EMD electrode surface during cycling.

**Figure 7:**
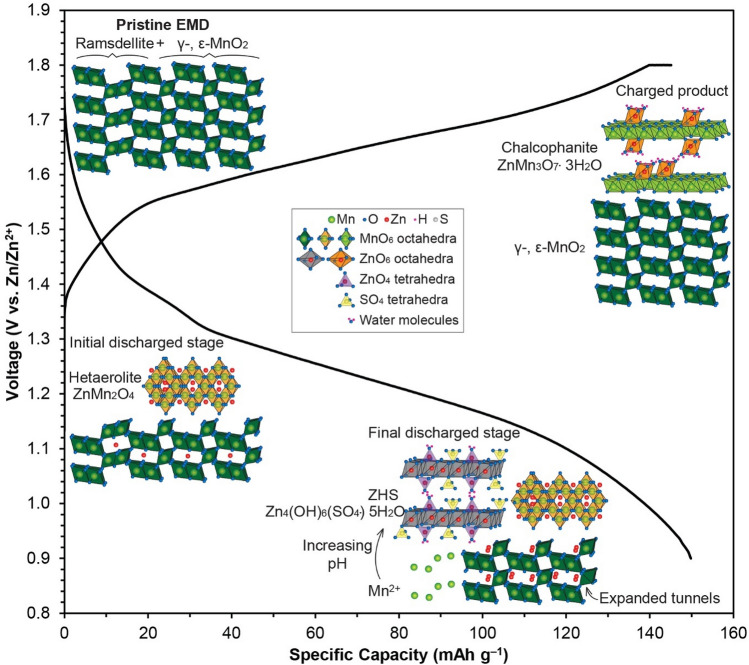
Schematic illustration of the proposed charge/discharge process for EMD.

The results indicate that during the early discharge stage (Fig. 7), a portion of EMD undergoes transformation to spinel-type ZnMn_2_O_4_, while Zn^2+^ ions are also inserted into the (1 × 2) tunnels of γ-/ε-MnO_2_ to form tunnel-type γ/ε-Zn_x_MnO_2_ at early depths of discharge (Fig. 4e). The structure of γ/ε-Zn_x_MnO_2_ further expands, as more Zn^2+^ ions are added into vacant tunnels, until the end of discharge. During the final discharge stage, disproportionation of intercalated Zn_x_MnO_2_ occurs which causes ZHS formation. Upon charging, tunnel-type γ/ε-Zn_x_MnO_2_ reverts almost entirely to the original γ- / ε-MnO_2_, while dissolved Mn^2+^ is redeposited with hetaerolite as layer-type chalcophanite.

## Conclusions

In this study, the electrochemical behaviour and structural changes for electrolytic manganese dioxide (EMD), utilized as a potential positive electrode for Zn-ion batteries (ZIBs), have been investigated. The initial EMD powder is composed of about 53% ε-MnO_2_, 34% ramsdellite, and 13% γ-MnO_2_. ZIBs using EMD have a multi-step reaction mechanism, which was confirmed by rotating ring-disk electrode (RRDE) analysis, X-ray diffraction (XRD), and electron microscopy. During discharge, Zn^2+^ ions are either intercalated to form spinel-type ZnMn_2_O_4_ or inserted into tunnels of MnO_2_ to form tunnel-type Zn_x_MnO_2_. The latter is unstable and once the saturation point for Zn^2+^ intercalation in the MnO_2_ tunnels (Zn_0.5_MnO_2_) is reached, Zn_0.5_MnO_2_ is disproportionated which results in zinc hydroxide sulfate (ZHS) formation. Electron microscopy and XRD analysis provided evidence of reversible ZHS formation and Zn^2+^ de-insertion. However, Mn^2+^ is electrochemically deposited back on hetaerolite to form chalcophanite. During cycling, the original ramsdellite portion of EMD disappears while layered chalcophanite gradually covers the electrode surface during charging. The electrochemical and chemical reactions are as follows:

During discharge:

**Table:** 

$(\mathrm{A}) \,\, {2\mathrm{MnO}}_{2}+{\mathrm{Zn}}^{2+}+{2\mathrm{e}}^{-}\to {\mathrm{ZnMn}}_{2}{\mathrm{O}}_{4}$	Hetaerolite formation
$(\mathrm{B})\,\, {\mathrm{MnO}}_{2}+{\mathrm{xZn}}^{2+}+{2\mathrm{xe}}^{-}\to {{\upgamma ,\upvarepsilon -\mathrm{Zn}}_{\mathrm{x}}\mathrm{MnO}}_{2}$	Zn^2+^ insertion
$(\mathrm{C}) \,\, 2{{\mathrm{Zn}}_{0.5}\mathrm{MnO}}_{2}+{2\mathrm{H}}_{2}\mathrm{O}\to {{\mathrm{Zn}}^{2+}+\mathrm{Mn}}^{2+}+{\mathrm{MnO}}_{2}+{4\mathrm{OH}}^{-}$	Disproportionation
$(\mathrm{D}) \,\, 4{\mathrm{Zn}}^{2+}+{6\mathrm{OH}}^{-}+{\mathrm{SO}}_{4}^{2-}+5{\mathrm{H}}_{2}\mathrm{O}\to {\mathrm{Zn}}_{4}{\left(\mathrm{OH}\right)}_{6}\left({\mathrm{SO}}_{4}\right)\bullet 5{\mathrm{H}}_{2}\mathrm{O}$	ZHS formation

During charge:

**Table:** 

$(\mathrm{E}) \,\, {{\mathrm{Zn}}_{\mathrm{x}}\mathrm{MnO}}_{2}\to {\mathrm{MnO}}_{2}+{\mathrm{xZn}}^{2+}+{2\mathrm{xe}}^{-}$	Deintercalation of Zn^2+^
$(\mathrm{F})\,\, \mathrm{ Zn}{\mathrm{Mn}}_{2}{\mathrm{O}}_{4}+{\mathrm{Mn}}^{2+}+{6\mathrm{H}}_{2}\mathrm{O}\to {\mathrm{ZnMn}}_{3}{\mathrm{O}}_{7}\bullet {3\mathrm{H}}_{2}\mathrm{O}+{6\mathrm{H}}^{+}+{4\mathrm{e}}^{-}$	Chalcophanite formation
$(\mathrm{G}) \,\, {\mathrm{Zn}}_{4}{\left(\mathrm{OH}\right)}_{6}\left({\mathrm{SO}}_{4}\right)\bullet 5{\mathrm{H}}_{2}\mathrm{O}+{6\mathrm{H}}^{+}\to 4{\mathrm{Zn}}^{2+}+{\mathrm{SO}}_{4}^{2-}+{11\mathrm{H}}_{2}\mathrm{O}$	ZHS dissolution

## Methods

### Preparation of EMD electrodes

All chemicals were used as received without further purification. EMD was obtained from Borman Specialty Materials (formerly Tronox).

In order to obtain a sufficient amount of cycled material for characterization, highly loaded EMD electrodes were prepared using a proprietary slurry cast method described elsewhere^50^. The mass loading of EMD was approximately 30–35 mg cm^–2^ on a graphite foil current collector.

### Electrochemical measurements

For rotating ring-disk electrode (RRDE) experiments, a Pine Research rotator (model: AFMSRCE), rotating electrode controller, and fixed disk RRDE tip were used. The working RRDE tip consisted of a 4.57 mm diameter glassy carbon electrode and a Pt ring electrode with a 180 μm gap between them. The collection efficiency of this geometry is 22%. The ink composition contained 75 µL Nafion, 7.5 mg carbon black, and 7.5 mg of MnO_2_ (EMD) in 5 mL of 20% v/v of isopropyl alcohol (IPA). The ink (200 µL) was dropped on the disk electrode several times. The counter electrode was a Pt wire, while the reference electrode was Hg/HgO. The RRDE assembly was operated at 900 rpm with Ar saturation of the electrolyte. The disk electrode was scanned from OCV to 0.9 V at 1 mV s^−1^, while the Pt ring was kept at 1.91 V to ensure that any soluble Mn^2+^ species were oxidized back to Mn^4+^. All potentials in this study are referenced to Zn/Zn^2+^, obtained by adding the measured values to 0.86 V (V_Hg/HgO_ = 0.098 V and V_Zn/Zn_^2+^  =  − 0.762 V vs. NHE).

Zinc sulfate heptahydrate (ZnSO_4_·7H_2_O) was purchased from Sigma-Aldrich for use in the electrolyte. Deionized water (DIW) was used to rinse electrodes and prepare electrolytes.

All electrochemical cells were assembled using a homemade plate design comprising a rubber gasket sandwiched between two acrylic plates^50^. The acrylic plates were bolted together and housed the electrode stack (negative/separator/positive). The electrode stack was compressed together between Ti plates by external screws (torque of 2 in-lb) which also served as electrical connections. Zn foil (from McMaster-Carr) was used as the negative electrode and three layers of filter paper (Whatman #1) soaked with 1 M ZnSO_4_ were used as the electrolyte and separator between the EMD and Zn electrodes. Electrochemical measurements were carried out with Biologic SP-300 and VSP-300 potentiostats. During the charging process, the batteries were held at 1.8 V for an additional 2 h or until the current density dropped to 8 µA mg^–1^, whichever came first.

### Materials characterization

The morphologies and compositions of the EMD samples were characterized using a scanning electron microscope (Tescan Vega3 SEM), coupled with an energy dispersive X-ray (EDX) spectrometer. X-ray diffraction (XRD) analysis was performed using a Rigaku Ultima IV diffractometer with monochromatic Cu Kα X-radiation (wavelength equal to 1.54 Å) at a scan rate of 5° min^–1^. Transmission/scanning transmission electron microscopy (TEM/STEM), X-ray microanalysis, and selected area electron diffraction (SAED) were performed using a JEOL JEM-ARM200CF TEM/STEM operating at an accelerating voltage of 200 kV. TEM/STEM samples were prepared by scraping the electrode surface and placing the residue into 1 mL of IPA. The suspension was sonicated for 30 min and one or two drops were placed onto a lacey carbon TEM grid. Nitrogen adsorption isotherms were measured at 77 K using a Micromeritics ASAP 2020 system. The EMD powder was outgassed at 200 °C for 12 h prior to any measurements. The BET method was used to measure the specific surface area of the sample at a relative pressure ranging from 0.05 to 0.30.

## Supplementary Information

Supplementary Information.
