# Supplementary material for: Reaction mechanisms for electrolytic manganese dioxide in rechargeable aqueous zinc-ion batteries
Source: Sci Rep. 2021 Oct 21;11:20777. doi: 10.1038/s41598-021-00148-2 (PMC8531032; doi:10.1038/s41598-021-00148-2)
Supplement: Supplementary file 1 — Supplementary Information. [file 41598_2021_148_MOESM1_ESM.pdf]

SUPPORTING INFORMATION

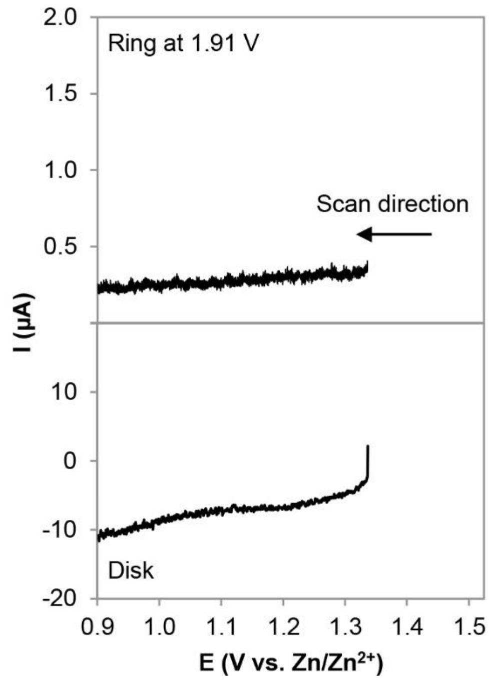

Figure S1. RRDE profiles of hetaerolite recorded at 1 mV s^{-1} in an Ar-saturated 1 M $ZnSO_4$ solution, stirred at 900 rpm with the Pt ring maintained at 1.91 V vs Zn/Zn^{2+}.

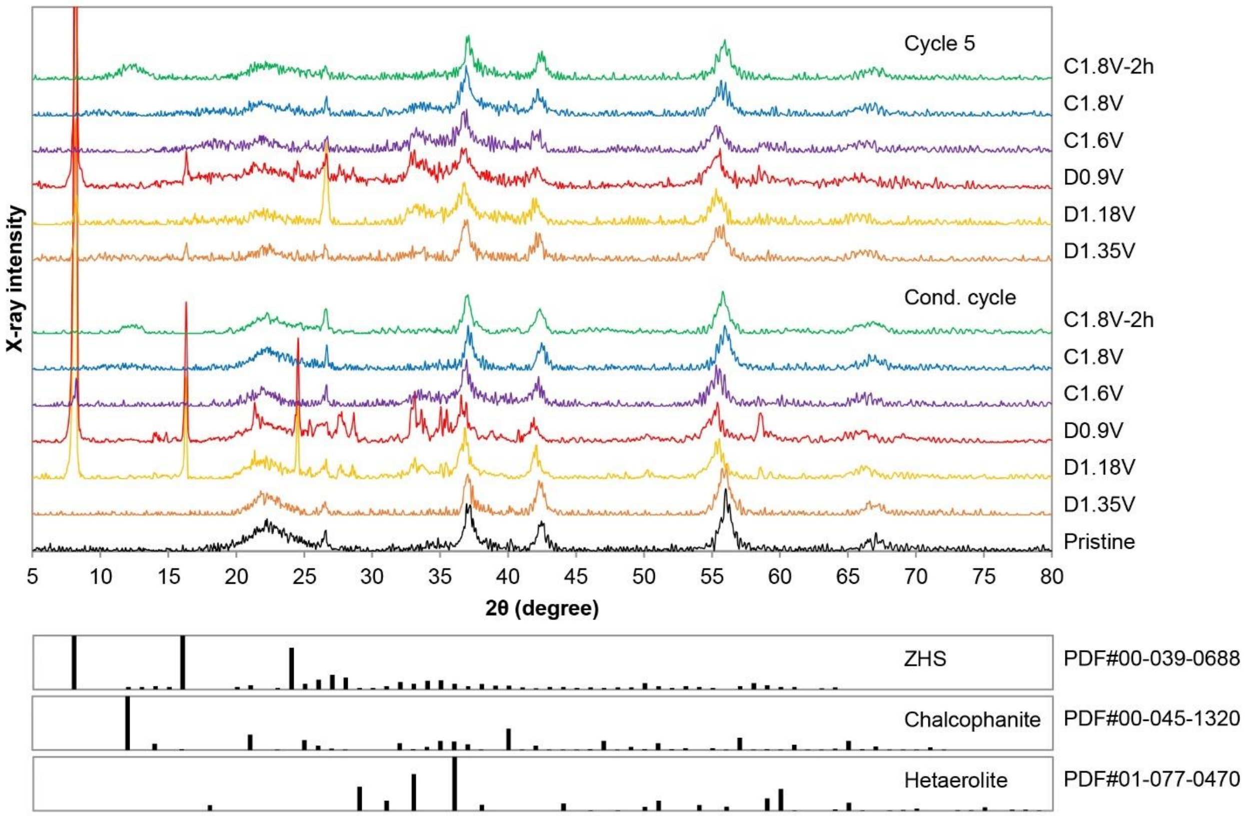

Figure S2. XRD patterns of EMD electrodes at different potentials during GCD tests with the conditioning cycle at 0.5 mA cm^{-2} and the 5th cycle at 1 mA cm^{-2}. Reference PDF cards for ZHS, chalcophanite, and hetaerolite are included at the bottom of the XRD patterns. Discharge and charge processes are denoted as D and C, respectively.

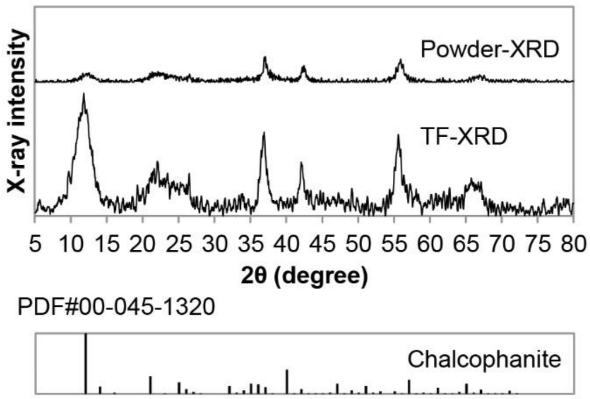

Figure S3. Powder XRD and TF-XRD patterns of the EMD electrode after CV cycling at 1.8 V for 2 h. The electrodes were washed with 1 mM AA. The PDF card for chalcophanite is included.

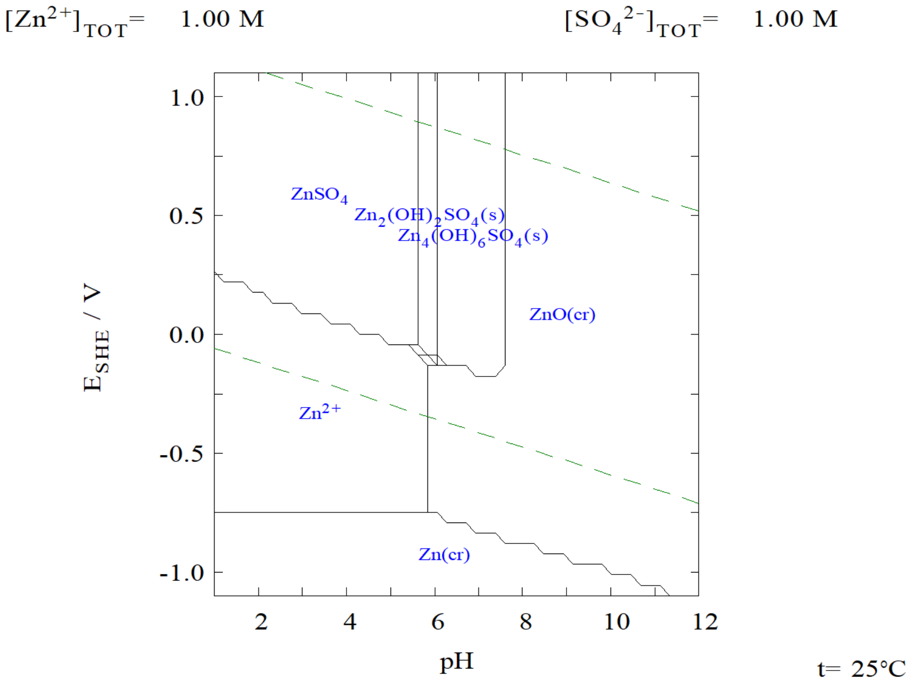

Figure S4. Pourbaix diagram for $ZnSO_4$ at ion concentrations of 1.00 M.

Table S1. Indexed d-spacings for the SAED pattern in Figure 5a and d-spacings from the PDF card for hetaerolite (PDF#01-077-0407)

Measured d-spacing (Å)	Hetaerolite $ZnMn_2O_4$ d-spacing (Å)	(hkl)
4.87	4.86	(101)
2.93	3.04	(112)
2.74	2.71	(103)
2.44	2.46	(211)
2.43	2.43	(202)
2.01	2.02	(220)
1.80	1.80	(204)
1.66	1.68	(312)
1.56	1.56	(321)
1.55	1.52	(224)
1.22	1.22	(404)
1.20	1.20	(325)
1.10	1.10	(415)
1.00	1.00	(514)

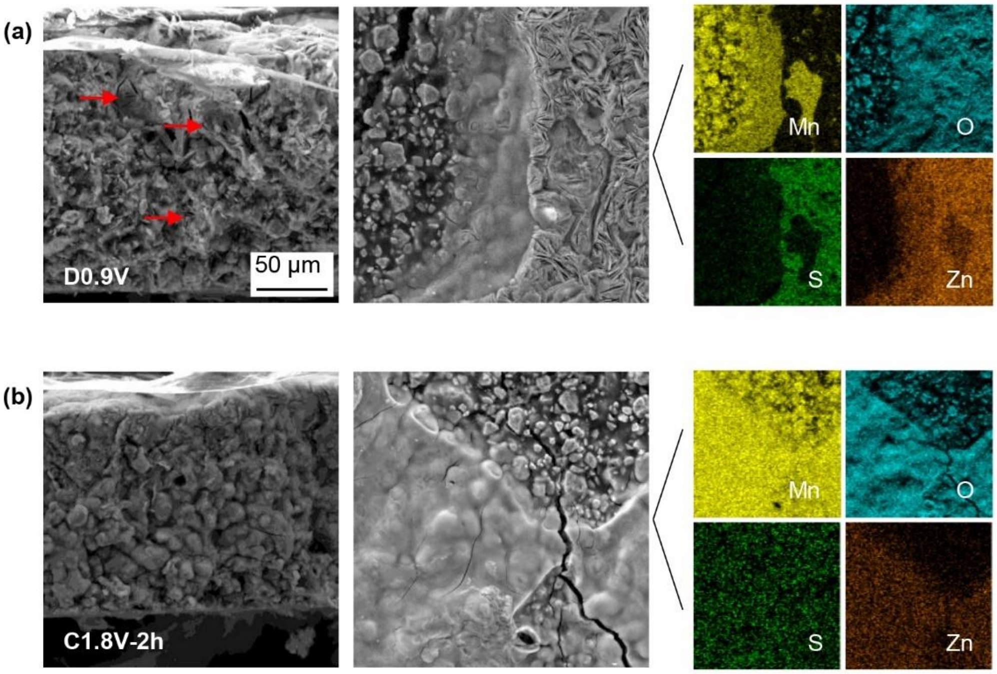

Figure S5. SEM SE images and EDX maps of EMD electrodes after 50 cycles: **(a)** After discharging to 0.9 V and **(b)** after charging and holding at 1.8 V. From left to right: cross section image, plan view image, and corresponding EDX maps. The red arrows indicate ZHS regions.
